# Studies on the electrical and optical conductivity of barium-nickel ferrite nanoparticles doped with Zn

**DOI:** 10.1186/s11671-024-04180-9

**Published:** 2025-01-06

**Authors:** Sadiq H. Khoreem, A. H. AL-Hammadi

**Affiliations:** 1 Department of Optometry and Vision Science/Faculty of Medical Sciences, Al-Razi University, Sana’a, Yemen; 2 Center of Research and Studies, Amran University, Amran, Yemen; 3 Physics Department/Faculty of Science, Sana’a University, Sana’a, Yemen

**Keywords:** Optical properties, Band gaps, Optical conductivity, Energy storage, Nano barium ferrite

## Abstract

The study highlights the significant effects of Zn ions concentration on the optical properties of BaNi_2-x_Zn_x_Fe_16_O_27_ ferrites, emphasizing the tunability of the band gap through Zn doping and explores their potential to enhance their optical properties. The barium-nickel ferrite powder, with the composition BaNi_2−x_Zn_x_Fe_16_O_27_, was synthesized using the ceramic method. The effects of Zn doping were analyzed using X-ray diffraction (XRD) and UV‒visible (UV–Vis) spectroscopy. XRD confirmed a pure single-phase W-type hexagonal structure, with an increase in both grain size and lattice constant proportional to the Zn content. The optical properties were assessed through UV‒visible spectroscopy, revealing an increaseing of the band gap with increasing Zn concentration, confirming material’s semiconducting behavior.All optical constants, exhibited consistent variation with increasing Zn substitution.. Additionally, both electrical and optical conductivities increased with rising photon energy, while the conductivity peak decreased with higher Zn content. The electric susceptibility was found to decrease as Zn concentration increased. The results indicate that Zn doping leads to significant changes in lattice parameters, crystallite size, and bandgap energy, suggesting potential applications in optoelectronics, photonics devices, and energy storage."

## Introduction

Barium W-type hexaferrites, in particular, have attracted significant interest because of their unique electrical and optical properties that make them prime contenders for magnetic storage, sensors, and biomedical devices. Their small bandgaps also raise their profile for applicability in photovoltaic devices, although many of the bandgap states are indirect and problematic for charge carrier dynamics [1–5]. Enhanced performance in photovoltaic contexts has prompted research into doping strategies to modify the electrical and optical properties of these materials [6]. Among the doping candidates, zinc has recently been highlighted because of the beneficial effects of its incorporation on the barium hexaferrite matrix, making the system more efficient for light absorption and other advanced photovoltaic applications [7, 8]. Doping zinc (Zn) into barium ferrites can improve their photovoltaic efficiency by enhancing their key electrical and optical properties. The research carried out by Ismael et al. (2021) has already amply described how Zn doping affects certain parameters that control these fundamental bulk properties. Doping with Zn has been confirmed to lower the “activation energy” (the energy required for a process to occur) for the hopping of electrons between Fe3⁺ and Fe2⁺ ions. [9]. This lowered activation energy not only increases electrical conductivity (a key property for applications like electromagnetic interference shielding and microwave absorption) but also enhances the "optical conductivity" of the material—how well it conducts electrical energy when undergoing a change in a light signal. This increase in optical conductivity has been attributed (by Mustafa, Ghulam, et al.) to a lower energy bandgap that the material has after doping with Zn [10].

In Ba-W ferrites that were made using the ceramic method, divalent metal ions either occupied octahedral sites or prevented grain formation by taking the place of tetrahedral Zn2⁺ ions. Nickel ferrites typically prefer octahedral sites, whereas zinc ferrites tend to occupy tetrahedral sites, influencing the magnetic properties of the material. [11]. In zinc-substituted Ba(Ni_2−x_Zn_x_)Fe_16_O_27_ ferrites, Khoreems et al. reported that BaNi-W exhibited very low permeability. They also reported an increase in permeability alongside a decrease in the ferromagnetic resonance (FMR) frequency with increasing Zn content. [12, 13]. Infrared spectral analysis of BaNi₂ − ₓZnₓFe₁₆O₂₇ ferrite over the 400–4000 cm^⁻1^ range revealed two distinct bands. The refractive indices, absorption coefficients, and reflectance spectra for different Zn doping levels were calculated. The results indicated that increasing the Zn content led to increased absorption capacity, decreased refraction, and changes in the refractive index of the material, altering its optical properties. [14]. The interactions between ions at tetrahedral and octahedral sites primarily influence the electric and magnetic properties of ferrites. Ni2⁺ and Fe3⁺ occupy octahedral sites, while Zn2⁺ and Fe3⁺ occupy tetrahedral sites in our barium nickel ferrite samples. The substitution of Zn2⁺ ions (which have a much smaller ionic radius than Ni2⁺ ions) for Ni2⁺ ions results in a lowering of the dielectric constant and the tangent of the dielectric loss over the whole frequency range (1–3 MHz) for our samples. The enhanced optical properties of our Zn-doped barium‒nickel ferrite make these materials suitable for a variety of applications and devices, such as high-frequency electronic components and advanced photonic devices. [15, 16]. This research primarily involved an investigation into how the optical properties of barium nickel ferrite nanoparticles (BaNi_2−x_Zn_x_Fe_16_O_27_) are affected by doping with zinc (Zn) at different concentrations. The aim in optimizing the materials for potential use in photovoltaic devices necessitated obtaining a semiconducting material with well-defined optical properties. Therefore, the synthesis of the nanoparticles was performed using the so-called ceramic method. Structural and optical analyses of the resulting nanoparticles enabled an examination of how well the material could perform as a photovoltaic device. The study concluded that the optical property of the material could be optimized by varying the Zn doping concentration.

## Materials and methods

This study’s experimental design was carefully devised to achieve the primary goal of synthesizing Zn-doped barium–nickel ferrite (BNF) nanoparticles. It also allows a systematic investigation of their properties and a thorough characterization of the effects of Zn doping.

### Synthesis

**Fig. 1:**
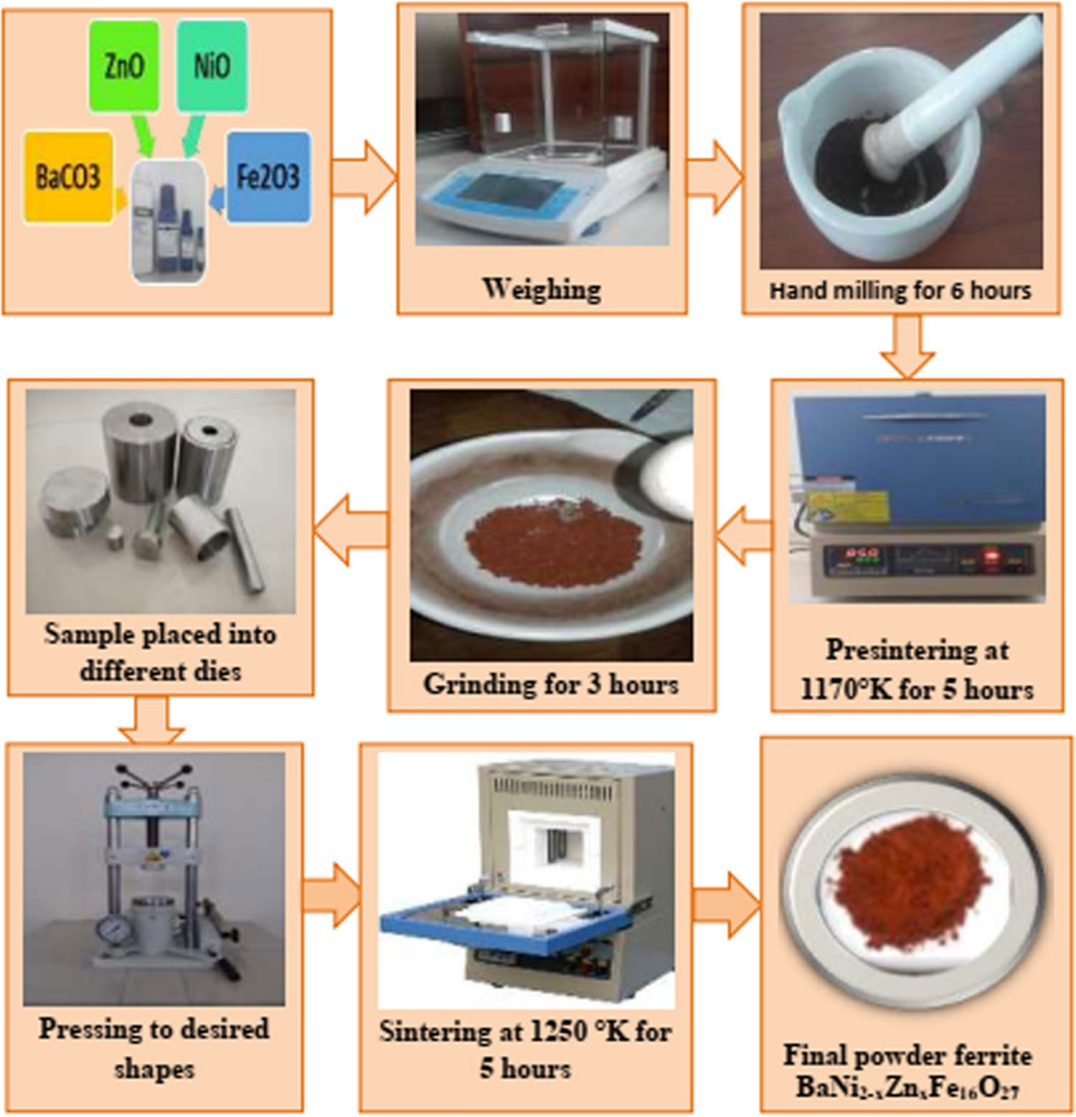
Flow chart of the ferrite sample preparation technique via the usual ceramic method

The ceramic method was used to synthesize zinc-substituted barium-nickel ferrites (BaNi_2−x_Zn_x_Fe_16_O_27_, where x = 0.0 ≤ 2.0). To produce the desired compositions, the raw materials—BaCO_3_, NiO, ZnO, and Fe_2_O_3_—were measured accurately according to their stoichiometric ratios. The mixture was then ground in an agate mortar for six hours—a step that’s crucial for ensuring the uniformity and proper blending of the starting materials. Six hours is the right amount of time; the unsaturated time is poor and leads to non-discrete phase formations. Three hours is too short; at four hours (the amount of time used in some previous study) steps forward were found toward this mixture being sintered in a furnace for two hours at 700 °C to gain some sort of medium-compact form (poor compaction leads to a piece that has few magnetically active sites). [16–18]. After presintering, the ferrites were reground and pressed into pellet form using a hydraulic press.These pellets were then sintered at 1250 K for five hours to promote complete phase formation and crystallization. The slow cooling of the pellets to room temperature at a controlled rate minimized internal stresses and ensured uniformity in the final product. The samples were then pulverized into fin powder. A schematic of the fabrication process is presented in the Fig. 1.

### Characterization

To thoroughly investigate the effects of Zn doping on the BNF nanoparticles. A suite of advanced characterization techniques was employed:

1. X-ray diffraction (XRD): XRD analysis was conducted via a Shimadzu EDX-720 X-ray diffractometer with Cu Kα radiation (λ = 1.5406 Å). The XRD patterns were collected over a 2θ range of 5°–75° at a step size of 0.02°/s to determine the crystal structure, phase purity, and lattice parameters of the synthesized material.
2. UV‒Visible (UV‒Vis) Spectroscopy: The optical properties of the Zn-doped BNF nanoparticle powder were uniformly dispersed in double distilled water in the presence of HCl (0.3 N), and the particles were characterized via Analytic Jena (SPECORD 200 model) and a double-beam UV‒visible spectrometer with a quartz cell (1 cm path length). The absorption spectra were recorded at room temperature in the wavelength range of 200–800 nm. Tauc plots were used to estimate the optical band gaps, allowing us to assess the impact of Zn doping on the material’s electronic structure and light absorption capabilities.

## Results and discussion

### XRD analysis

**Fig. 2:**
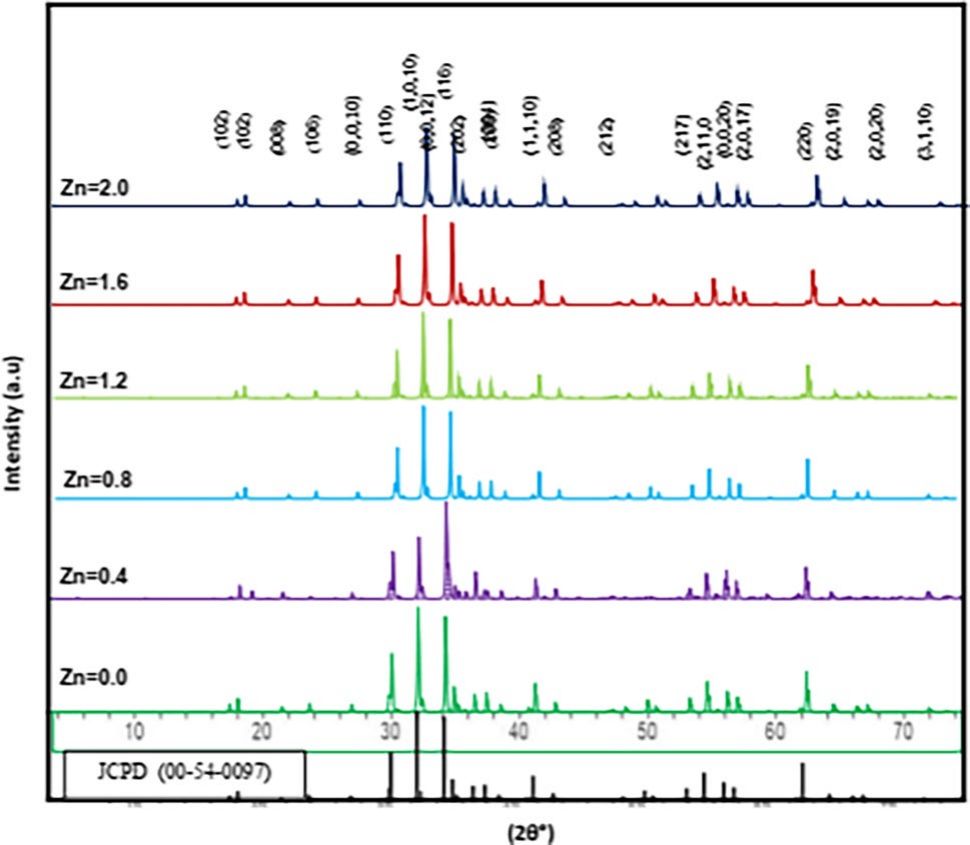
XRD patterns of the Zn^2+^ concentration doped for BaNi_2-x_Zn_x_Fe_16_O_27_

X-ray diffraction (XRD) analysis was conducted to determine the phase composition and crystalline structure of the synthesized barium-nickel ferrite (BaNi₂₋ₓZnₓFe₁₆O₂₇) nanoparticles. The XRD patterns for samples with varying Zn concentrations (x = 0.0, 0.4, 0.8, 1.2, 1.6, 2.0) were recorded over a 2θ range of 5° to 75° via Cu Kα radiation (λ = 1.5406 Å) and are presented in Fig. 2. All concentrations of Zn resulted in a single-phase W-type hexagonal ferrite structure. The characteristic diffraction peaks observed at 2θ values of 30.22°, 31.88°, 32.29°, 34.45°, 35.50°, 36.90°, 55.21°, 57.59°, and 62.92° correspond to the (110), (112), (1010), (116), (202), (204), (2110), (2016), and (220) planes, respectively, which are consistent with the standard JCPD (00–54–0097) for hexagonal barium ferrite. The XRD patterns showed no secondary phases, indicating that the synthesis route was effective.

The crystalline structure reveals subtle shifts in the characteristic peak, which retain their positions within the hexagonal ferrite framework as the Zn concentration increases, confirming that Zn^2^⁺ ions have been successfully incorporated into the lattice. The well-defined peaks suggest that the nanoparticles exhibit high crystallinity, which is essential for their magnetic and dielectric properties. The most obvious effect of substituting Zn for Ni was a shift of the (1010) and (202) peaks. The (1010) peak shifted 5.05° toward lower 2θ angles, and the (202) peak shifted 4.80° in the same direction. Because this was a straight substitution of Zn for Ni, we can use the shifts in these peaks to make a reasonable guess about the amount of Zn that is actually incorporated into the hexagonal structure. [19]. The substitution of Ni^2^⁺ with Zn^2^⁺ causes an expansion of the unit cell, leading to an observed shift in peak positions.

The lattice parameters (a and c) were calculated from the XRD data via relation (1) and the geometry of the hexagonal structure. [11, 20].

1 $$\frac{1}{{d}_{hkl}^{2}}=\frac{4}{3}\frac{{h}^{2}+{hk}+{k}^{2}}{{a}^{2}}+\frac{{L}^{2}}{{C}^{2}}$$

where h, k, and l are the Miller indices of the diffraction plane and where *d*ℎ*kl* is the spacing between the diffraction planes.

As expected, both lattice parameters increase with increasing Zn content, confirming the increase of the lattice constant. This is attributed to the incorporation of Zn, which has a larger ionic radius (0.074 nm) compared to Ni ions (0.069 nm). Furthermore, the W-type structure is identifiable when the ratio of the hexagonal lattice parameters c/a is between 5.33 and 5.55. [14, 21]. The c/a ratios of our samples, as shown in Table 1, align well with this range, suggesting the formation of a W-type hexagonal structure. Table 1. Provides a summary of the value lattice parameters along with the corresponding c/a ratios.

**Table 1:** Lattice constant, c/a ratio, Crystallite Size, Volume cell, and Dislocation density in Zn-doped BNF nanoparticles

Zn Concentration	Lattice Parameter (Å)		ratio	Crystallite size (nm)	Volume cell (Å^3^)	Dislocation density (nm^−2^)
(x)	a	c	c/a	D	*V*_*cell*_	δ
0.0 (undoped)	5.975	32.735	5.478	36.50	1012.089	7.506E-4
0.4	5.976	32.742	5.478	35.181	1012.644	8.079E-4
0.8	5.978	32.759	5.479	35.531	1013.848	7.921E-4
1.2	5.982	32.785	5.480	35.814	1016.0115	7.796E-4
1.6	5.986	32.877	5.492	36.376	1020.225	7.557E-4
2.0	5.998	32.899	5.484	36.664	1025.005	7.439E-4

The progressive shift in peak positions with increasing Zn content clearly indicates the doping effect and the resulting lattice expansion. These shifts are crucial, as they confirm that Zn effectively replaces Ni in the lattice, thereby altering the structural parameters of the material.

In addition to the lattice constant changes, the crystallite size was also determined from the XRD data via Scherrer’s formula [11, 12].

2 $$D = \frac{\user2{K}\lambda}{{\beta \,\user2{hkl}\,\textit{cos} \,\theta }}$$

where D represents the crystallite size, λ is the wavelength (1.5406 Å) X-ray diffraction, θ is the angle diffraction of Bragg’s, k is the factor shape, which is typically 0.9 for a hexagonal structure, and βhkl is the full width at half maximum (FWHM) of the diffraction peak.

The grain size was found to vary with the Zn concentration, as shown in Table 1. Initially, the crystallite size decreased slightly with the initial introduction of Zn (x = 0.4) and then increased as the Zn concentration continued to rise. This trend suggests that Zn doping initially disrupts the crystal growth process, but with higher doping levels, the crystal structure stabilizes, leading to an increase in the crystallite size. However, as the Zn content increases further, the crystallite size begins to increase, indicating the stabilization of the crystal structure.

The hexagonal structure unit cell volume for the BaNi_2-x_Zn_x_Fe_16_O_27_ferrites was computed from the relation [20, 21]:

3 $$V_{{cell}} = a^{2} c\,\textit{Sin} \,(120^{^\circ } )$$

The cell volume increases with concentration of Zn. In this case, the increase in cell volume can be attributed to the difference in ionic radii of the dopant (Zn^2+^) and the host (Ni^2+^) ions. With a larger ionic radius, the incorporation of Zn^2+^ into the lattice expands the structure. The gradual increase in the cell volume with concentration of Zn serves, therefore, as a direct indicator of the ionic substitution taking place.

Zn^2+^ has a larger ionic radius than Ni^2+^. This might make Zn^2+^ diffuse into grain boundaries more easily and, in effect, form a very thin insulating layer around the grains, which could be influencing the grain growth and, thus, the properties of the material. An influence of this kind is more than a little strange to contemplate, especially considering that both Ni and Zn are d-band metals and have similar electronegativities. Yet, there’s no arguing with the numbers: as we add Zn^2+^, both the fundamental lattice parameter "a" and the volume of the unit cell increase concomitantly. Meanwhile, the average crystallite size shows a slightly weird progression, going from about 36.5 nm at x = 0.0 down to 35.18 nm at x = 0.4 and then up to about 36.7 nm at x = 2.0.

The dislocation density (δ) was evaluated with the following equation [21, 22]:

4 $$\delta =\frac{1}{{{\varvec{D}}}^{2}}$$

Here, D is the average size of the crystallites, and being inversely proportional to it, dislocation density tends to afford a larger value for a smaller crystallite size. The dislocation density values we found for our samples at Zn = 0. 4 site doping were consistently high and ranged from about 7.9 × 10^–4^ to 7.3 × 10^–4^, as listed in Table 1.

### Optical measurements

**Fig. 3:**
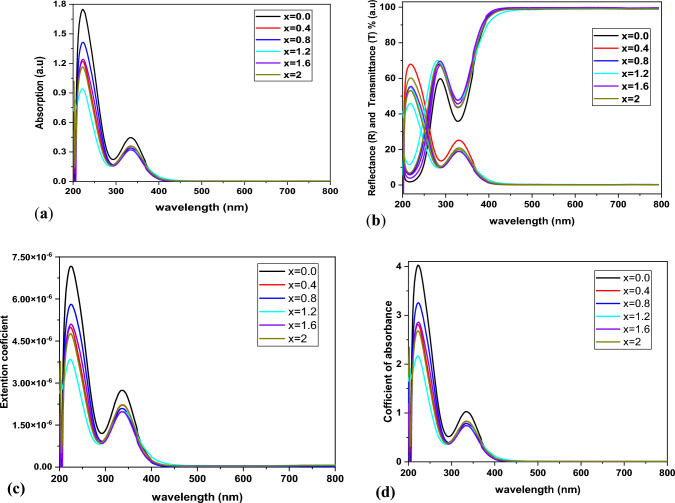
The spectrum behaviour for synthesis sample as: **a** absorption, **b** transmission with reflectance, **c** coefficients of extinction, and **d** coefficient of absorption

**Fig. 4:**
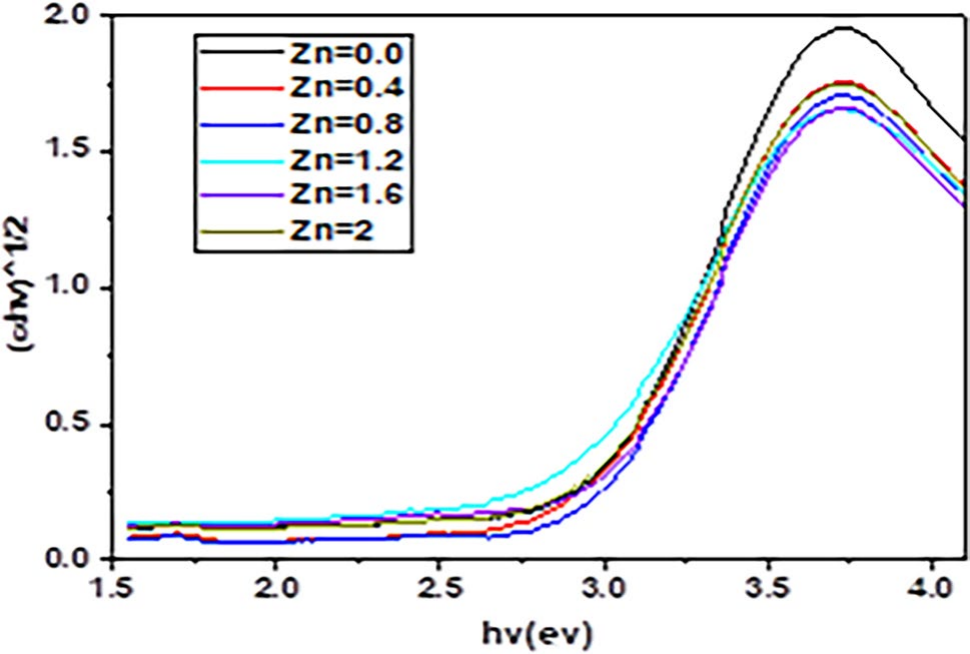
Tauc plots for BaNi_2-x_Zn_x_Fe_16_O_27_

The optical properties of Zn-doped barium-nickel ferrite (BaNi₂₋ₓZnₓFe₁₆O₂₇) nanoparticles were analyzed via UV‒visible (UV‒Vis) spectroscopy. The analysis focused on the absorption spectra and the corresponding bandgap energies to understand how Zn doping influences these properties. The UV‒Vis absorption spectra of the samples recorded in the wavelength range of 200–800 nm revealed significant changes in the absorption behavior as a function of the Zn concentration, as displayed in Fig. 3a. As the Zn content increased, a noticeable blueshift in the absorption edge was observed, indicating that the optical absorption of the material moved toward shorter wavelengths. The absorption edge shifts significantly between the undoped sample (x = 0.0) and the highly doped sample (x = 2.0). This indicates that the bandgap of Ba-Ni increases with the concentration of Zn2⁺, and the material exhibits a more pronounced blueshift [23]. We observe that as Zn doping increases, not only does the bandgap blueshift, but also the appearance of the spectrum behaves differently in correlation with the concentration of Zn. The changes in Zn-doped samples are attributed to two main structural effects: the size of the crystallites and a blueshift that is significant in both amplitude and appearance [24]. When the material is doped with zinc (Zn), the size of the crystallites increases, which in turn affects the optical bandgap. From Fig. 4.a, it can be deduced that with the added charge carrier concentration from zinc, there is a movement of the absorption edge to higher energies with increasing doping. This indicates that the energy required to excite an electron from the valence band to the conduction band also rises with the added charge carriers from the Zn. These findings suggest that the optical bandgap increases with increasing Zn content. The highest absorbance seen at the Zn content of 334 nm in the visible range (and also at 234 nm in the ultraviolet region) suggests that the absorbance is decreased with an increase in the Zn content (and also the crystallite size), as observed in the UV–Vis images and optical density plot up to 0.434 seen at 260 nm. The results indicated that each sample was transparent at broader wavelengths (400 nm), with no absorption or scattering occurring in the nonabsorbing region (R + T = 1). At shorter wavelengths (λ = 400 nm), where absorption occurs, a deviation is noted (R + T < 1). Additionally, the edge of the transmitted light for the samples shifts slightly toward shorter wavelengths, suggesting that the energy gap widens as the Zn concentration increases. Notably, the transmittance generally increases with increasing wavelength across the spectrum from 200 to 400 nm, except for a significant decrease at approximately 350 nm.

For each sample, the transmission increases with increasing wavelength between 332–338 nm range until it attains saturation at extended wavelengths. This material has most absorption at 334 nm, making it well-suited for applications in reagents or solar cells. The samples are transparent in the visible and infrared regions of the spectra, with the highest transmission observed in these regions and the lowest in the ultraviolet spectrum. “Figure 3 shows that the absorption spectrum behaves differently from the transmission spectrum”.

Reflectance (R) was calculated via the transmittance (T) and absorbance (A) via the following relation [23]:

5 $${\text{R}}\left( \lambda \right) = {1} - {\text{T}}\left( \lambda \right) - {\text{A}}\left( \lambda \right){\text{R}}$$

Figure 3b shows the variation in the reflectance of the composite across the 200–800 nm wavelength range. The reflectance clearly decreases, as the Zn ions doping amount in the composite increases, while the reflectance-wavelength behaviour profile remains consistent, indicating minimum absorption in the near-infrared and visible regions. Reflectance (R) and Transmittance (T).

The absorption coefficient (α) is a critical parameter in evaluating the optical properties of ferrite materials. It provides insight into the material’s ability to absorb light at different wavelengths, which directly influences its potential applications in various optical and electronic devices. In this study, the coefficient of absorbency was calculated using the following equation [25].

6 $$\alpha = 2.303A/t$$

In this formula, A, is the absorbance, and t represent the sample diameters.

As shown in Fig. 3d, the maximum absorption coefficient occurs in violet light at 334 nm, with an absorption ratio of 30%. This means that at these wavelengths, we expect direct electronic transitions (high photon energy) to occur. In the ultraviolet region, absorption attains 70% at 270 nm, the point at which the scones form. For all ferrite samples, the absorption coefficient decreases with increasing wavelength and, at some point, it stabilizes and becomes almost constant. One of these notable invariants occurs in the visible region, with an absorbance ratio of around 13%.

As the wavelength (λ < 220 nm) approaches the ultraviolet region, the absorption coefficient decreases. This primarily arises from the absorption properties of zinc and its compounds. In this context, it is notable that the composition of a substance defines its ability to absorb light at certain wavelengths. Zinc is a strong candidate for use in composites, for instance, as a representative of these entities due to its strong extinction coefficients at many important optoelectronic wavelengths. Consequently, as composites containing zinc and its compounds are formed, light absorption emanating from them can be expected. This will lead to expected maximum transmittances of about 78% through layer-type composites under ambient conditions.

### Extinction coefficient (K)

The key optical property that reveals the quantity of light that is lost due to absorption and scattering at a given wavelength in a material is the extinction coefficient (K). It is essential for understanding how a given material interacts with electromagnetic radiation and is computed using the relation [26].

7 $$K = \alpha \lambda /4\pi .$$

The absorption coefficient (α) of a material is thus tied to its extinction coefficient, with the formulation indicating that the K value will always be equal to or greater than the α value. Figure 3c shows that the K value behaves similarly with respect to wavelength (λ) as the α value. In fact, it has a trend that is almost indistinguishable from that of the absorption coefficient, with both values reaching their maxima in the wavelengths around the ultraviolet (UV) region of the spectrum.

Moreover, the extinction coefficient decreases as the Zn concentration in the samples increases; this indicates that the sample absorbs and scatters less light. Hence, the extinction coefficient tends to be lower for the greatest Zn concentrations. While a low absorption coefficient means that less light is absorbed by the material, and a low scattering coefficient means that less light is scattered in the material, these two properties work together to achieve a low-extinction condition. Overall, this condition tends to be more favorable for high-performance devices because if light is going to be present in the material, it must be in a state where it is not lost through light absorption or scattering.

The same changes in structure and composition within the material influence both of these properties, especially the presence of Zn. The extinction coefficient is a comprehensive measure of light interaction. It was observed to decrease with increasing Zn content. This means that not as much light is being scattered or absorbed when Zn is present, which is a very good indicator for optical applications—particularly for the kinds of applications where you want to minimize light loss.

### Optical band gap

The optical band gap (Eg) is a critical parameter design for proper optoelectronic device operation, as it defines the energy range in which a material can absorb light. It was calculated using the following equation [27]:

8 $$\alpha = \frac{{A\left( {h\nu - Eg} \right)^{n} }}{{h\nu }}$$

where α is the optical absorption coefficient, A is a constant related to the probability of the electronic transition, Eg is the optical band gap, hν represents the photon energy, and n depends on the nature of the transition. For allowed direct or indirect transitions, n equals 1/2 or 2, respectively, while for forbidden direct or indirect transitions, n equals 3 or 3/2.

The optical bandgap energies (Eg) were calculated via Tauc plots, which involve plotting (αhν)^1/2^ against the photon energy (hν). In these plots, the linear portion is extrapolated to the energy axis to determine the band gap values as shown in Fig. 4.

As the Zn concentration increased from 0.0, to 2.0, the energy band gap values tended to increase. This expansion, as shown in Fig. 4, suggests potential quantum confinement effects, where smaller crystallite sizes yield discrete energy levels, thus increasing Eg. The results indicate that the bandgap energy increases progressively with increasing Zn doping concentration, as shown in Table 2. The undoped BNF sample has a bandgap of approximately 2.78 eV, whereas the Zn-doped samples have an increased bandgap, reaching 2.94 eV at the highest Zn concentration (x = 2.0). The changes in optical properties due to Zn doping are closely related to structural effects: lattice expansion and increase in the bandgap: Zn doping leads to lattice expansion, as larger Zn^2^⁺ ions replace smaller Ni^2^⁺ ions, causing relaxation of the crystal lattice. This expansion increases the energy needed for electronic transitions, resulting in a wider bandgap. Crystallite size and absorption shifts: Initially, Zn doping decreases the crystallite size, causing a blueshift in the absorption spectra due to quantum confinement effects. When the dopant concentration increases, so does the crystallite size, and with it, the optical bandgap. This size increase, especially in the samples doped with higher concentrations of Zn, suggests that at least a part of the optical bandgap is due to the influence of quantum confinement. This means the doped crystals (especially those with larger sizes) have a light emission mechanism at worthwhile “lower depths” in the conduction band—a part of the conduction band that has fewer available “free passengers” (electrons) to ride the light-emission train. Hence, a study by Guo et al. on the reason for the wide optical band gap in the ZnO|ZnS system attributes it in part to this condition (attributable to the size increase with the amount of Zn in the samples) [23], This is in line with the overall trend of band gaps blueshifting in nanoparticles and is significant for adjusting materials’ optical properties for certain applications [28, 29]. Almost all the Zn-doped samples exhibited a strong blueshift; that is, the band gap increased substantially. This shift is important because it suggests not just enhanced, but dominant, visible light photoactivity of the Zn-doped samples compared to their undoped counterparts. Normally, the band gap decreases (becomes less energy demanding) as particle size increases, an expected trend that obeys quantum bonding. [30]. The gathered information suggests that a higher concentration of Zn^2+^ in the system leads to a higher band gap. This directs one to conclude that the optoelectronic properties of this ferrite compound can be significantly influenced by the level of Zn doping. Moreover, this characteristic shift toward a higher band gap with higher Zn content in the system, accompanied by a significant and consistent blueshift (compared to other samples) in the optical properties of the Zn-doped samples, reinforces the idea that these materials might be especially suitable for use in solar cells.

**Table 2:** Optical band gap values of the BaNi_2-x_Zn_x_Fe_16_O_27_ ferrite composite

Zinc Concentration (x)	Band gap (ev)
0.0 (undoped)	2.78
0.4	2.89
0.8	2.79
1.2	2.91
1.6	2.93
2.0	2.94

Moreover, the powerful absorption of these materials at nearly 334 nm means they are prime potential candidates for UV light sensors and for use in photocatalytic applications in which UV light plays a driving role. Substituting zinc for other cations in the ferrite structure affects the electronic environment. Tetravalent cations (like Ti^4+^) can replace divalent cations (like Ni^2+^) in the lattice. Zn^2+^ ions can act like Ti^4+^ and replace Ni^2+^, but the local environment around the Zn^2+^ ions is different from that around divalent or tetravalent cations. Consequently, substituting Zn^2+^ for other cations in the ferrite structure affects the overall density of states and the band gap.

As the content of Zn in the material increases, so does the band gap. This might be because the energies of the band edges increase, and the effective masses of the electrons and holes decrease. The increase in band gap results in an increase in the energy corresponding to the absorption edge. This effect may enhance the use of these materials in optoelectronic devices. The substantial effect of zinc doping on the optical characteristics of BNF nanoparticles is clear. As the doping concentration of zinc increased, the absorption edges consistently shifted toward shorter wavelengths, indicative of a corresponding increase in the optical band gap. This observation certainly plays into the hands of those who would contend that using zinc, as opposed to an alkali or alkaline earth metal, in the formation of doped semiconductor nanoparticles is a good approach when one is seeking to achieve a material with a desired band gap. The speculation that using zinc will increase the optical band gap of the semiconductor nanoparticles to a value that exceeds what one can obtain with other commonly used cation dopants (e.g., using lithium or magnesium instead) would appear to be supported by the experimental evidence.

The values for the energy gap (Eg) of BaNi_2-x_Zn_x_Fe_16_O_27_ferrites fall within the moderate range of 2.78 eV–2.94 eV. Hence, they can be considered for optoelectronic applications that require intermediate-band gap materials, such as those involving visible light absorption and photocatalytic processes. In comparison with related materials, the Eg values of BaNi_2-x_Zn_x_Fe_16_O_27_lie well above those of BaFe_12_O_19_ and BaM ferrites, which have been shown to yield Eg values extending only up to ~ 2.5 eV. Luna’s group [31] and Asiri’s group [29] have reported values that range from 1.74 eV to 2.47 eV for BaFe_12_O_19_ and BaM ferrites, respectively. In contrast, lower than the Eg observed in Gd-Co-substituted BaM, the values are between 4.07 eV and 4.28 eV [32]. indicating a balance between structural order and electronic configuration. This makes them promising candidates for various optoelectronic applications, including those that operate within the visible and potentially UV-light ranges.

### Refractive index (n)

The materials to be used in integrated optical devices must have well-defined optical properties, and of these properties, the most crucial to the design of the device is the refractive index. The index of refraction (n) is deemed to be among the most fundamental and significant properties of optical materials due to its association with the local electromagnetic field within the material and the polarization of the electronic and ionic constituents of the material. We computed the refractive index using the connection we established between the electronic structure and the local field in the material using formula (9) [33].

9 $${\text{n }} = \, \left[ {\left( {{1} + {\text{R}}} \right)/\left( {{1} - {\text{R}}} \right)} \right] \, + \left[ {\left( {{4} \times {\text{R}}} \right)/\left( {{1} - {\text{R}}} \right)^{{2}} - {\text{k}}^{{2}} } \right]^{{{1}/{2}}}$$

where *R* denotes reflectance and k represents the extinction coefficient.

**Fig. 5:**
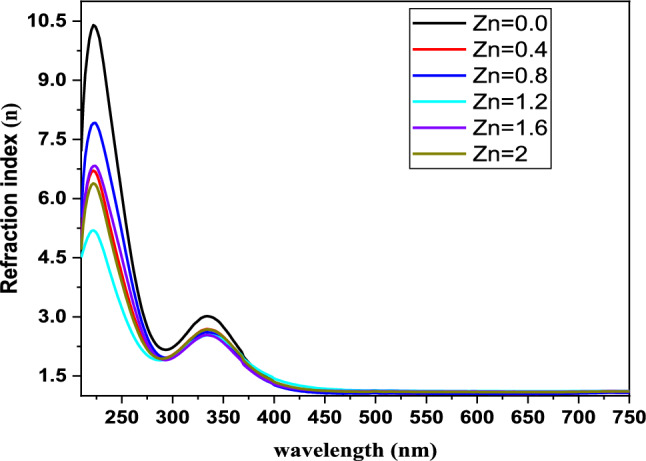
Refractive index for BaNi_2-x_Zn_x_Fe_16_O_27_ against wavelength

The development of optical materials and devices critically depends on the refractive index, which determines how light behaves when it encounters the material. The behavior of light at the external boundaries of a material, as well as the internal behavior of light inside a material, is solely controlled by the refractive index. As our study shows in Fig. 5, the refractive index tends to increase with wavelength and peeks at about 334 nm. However, after this point, the refractive index starts to drop while the wavelength keeps getting longer and moves deeper into the visible spectrum.

A key takeaway is the effect the concentration of zinc (Zn) nanoparticles has on the refractive index. When the Zn content goes up, the refractive index goes down. This drop in the refractive index shows that the addition of Zn is doing something to the optical properties of the material.

The links among photonic energy, zinc concentration, and fundamental optical properties such as the extinction coefficient and refractive index are important to understand. The concentration of Zn affects the basic materials’ performance in a photonic system, as shown by their increasing k and n when photonic energy increases, with Zn content as a variable. That relationship is essential for determining the basic suitability of the materials for optoelectronic applications, where a precise and accurate relationship among absorption, reflection, and transmission of incident light is necessary.

### Optical conductivity

Optical conductivity is an effective method for examining the atomic structure of materials. This property depends critically on the refractive index and absorption coefficient of the material under investigation. The connection between these two quantities and the material’s response to an electric field in the vicinity of the light waves reveals a great deal about the electronic structure of the material. The optical responses of the BaNi_2-x_Zn_x_Fe_16_O_27_ compound were measured for a variety of Zn concentrations (0.0 ≤ x ≤ 2.0) and used to calculate the optical conductivities for these various compositions at a variety of photon energies using the following equation [33]:

10 $${\sigma }_{opt}=\frac{\alpha nc}{4\pi }$$

The absorbing coefficient is represented by α, the refraction index by n, and the light velocity by c.

**Fig. 6:**
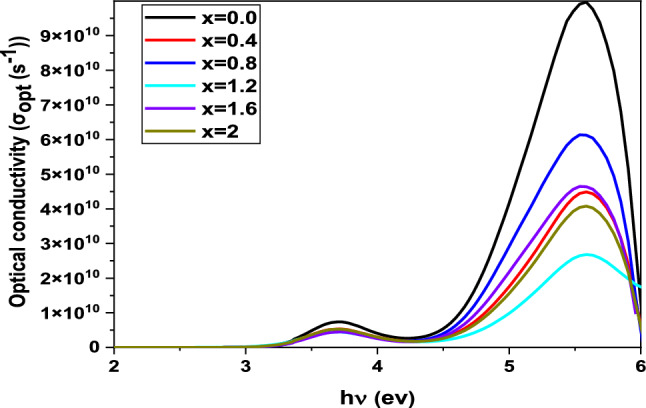
Optical conductivity with photon energy behaviour for BaNi_2-x_Zn_x_Fe_16_O_27_ ferrites

Figure 6 illustrates the optical conductivity of BaNi_2-x_Zn_x_Fe_16_O_27_ ferrites as a function of photon energy. The results indicate that optical conductivity starts at a low value and moves up along increases with the energy of incoming photons, beginning to rise significantly at 5 eV and reaching a a peak value at "5.5 eV" before coming down again. The optical conductivity is essentially a measure of how well a material can conduct electricity when exposed to light—in this case, visible and ultraviolet light. BaNi_2-x_Zn_x_Fe_16_O_27_ is a pretty good conductor when it is exposed to light of around 5.5 eV energy (visible/UV range) and does not conduct well when exposed to light of other energies.

Two distinct optical conductivity thresholds emerge, corresponding to different wavelength regions. These thresholds contribute to the two observed photoconductivity peaks, with the material showing higher absorbance at critical wavelengths in the visible and UV regions. The decrease in optical conductivity with increasing Zn concentration corresponds to widening of the bandgap. As the bandgap increases, fewer charge carriers are available for conduction, leading to reduced optical conductivity. This inverse relationship highlights the material’s potential for use in applications requiring precise control of electronic and optical properties, such as optoelectronics and photovoltaics. Moreover, the optical conductivity shows a direct link with the absorption coefficient. For our samples, the ultraviolet optical conductivity (σ_opt_) was found to be between 8.8 × 10⁹ and 1 × 10^11^ s⁻^1^, while in the visible region, it ranged from 9.1 × 10⁸ to 8.8 × 10⁹ s⁻^1^. These photosensitive elements inscribed on a glass substrate maintain their photosensitivity across both spectral ranges. Optical conductivity is directly influenced by the absorption coefficient, and this is plainly reflected in the optical conductivity values for the samples. In the ultraviolet wavelength range, the conductivity values are between 5 × 10^6^ and 9 × 10^10^ s^−1^, while in the visible range, they vary between 4 × 10^6^ and 5 × 10^6^ s^−1^, indicating that the samples maintain a high level of photosensitivity. These findings are consistent with previous studies. Moreover, an experimental determination of the electrical conductivity of the samples was made from expressions found in the same earlier work [34–36].

Additionally, the electrical conductivity of the samples was calculated using the relation from [35].

11 $${\sigma }_{e}=\frac{2\lambda {\sigma }_{opt}}{\alpha }$$

**Fig. 7:**
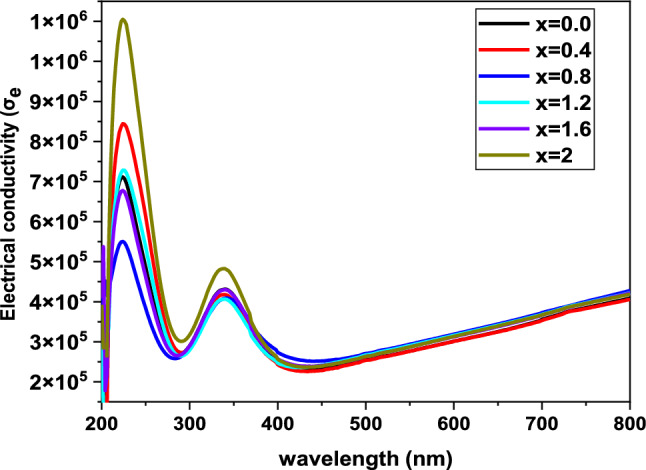
Electrical conductivity of BaNi_2-x_Zn_x_Fe_16_O_27_ ferrites as a function of wavelength

Figure 7, How electrical conductivity varies with wavelength provides key insights into the optical and electronic properties of BaNi_2-x_Zn_x_Fe_16_O_27_ ferrites. With increasing Zn concentration, the samples show a marked decrease in electrical conductivity. This trend can be understood in terms of the effect of Zn on the electronic structure of the materials; higher Zn content seems to reduce the mobility of charge carriers and thus to reduce electrical conductivity. Furthermore, the substitution of Ni ions by Zn, which has quite different electronic properties, may also play a significant role in influencing the conductivity of the materials.

Moreover, the dependence of electrical conductivity on wavelength shows a fascinating trend. At first, the electrical conductivity drops with the rising wavelength, inferring that the interactions of the material with the incoming photons are indeed less in this particular spectral region. However, upon surpassing an approximate threshold of 400 nm, the conductivity starts rising again. This shift could possibly be attributed to the absorption characteristics of the material at longer wavelengths, where the incoming photon’s energy is lower, and hence, the material could be more conductive due to a variance in the electronic transitions leading to a different augmented interaction of the material with the incoming photons. What this means is that the optical and electronic properties of Zn-doped ferrites are so closely linked that one can tune the conductivity of this material by adjusting its optical absorption. This has potential application in making devices that will work at specific wavelengths, like in the near-infrared. Understanding these effects could help us make more efficient devices. “BaNi_2-x_Zn_x_Fe_16_O_27_ ferrites show strong absorption in the near-infrared (NIR) region. There was tendency seen in the study for these particular materials to not only absorb but also manipulate the electrical conductivity, which is a very important property for semi-conductive magnetic materials to have”.

The electrical susceptibility of a dielectric material indicates its ability to polarize in response to an applied electric field. Higher electrical susceptibility means the material can polarize more effectively, thereby reducing the overall electric field within the material. The electrical susceptibility (${\chi }_{c}$) was calculated from the optical constants using the following relation [37].

$${\varepsilon }_{r}={\varepsilon }_{^\circ }+4\pi {\chi }_{C}={n}^{2}+{K}^{2}$$

12 $${\chi }_{c}=\frac{{n}^{2}-{k}^{2}-{\varepsilon }_{0}}{4\pi }$$

where ${\varepsilon }_{0}$ represents the dielectric constant, without a free carrier contribution.

**Fig. 8:**
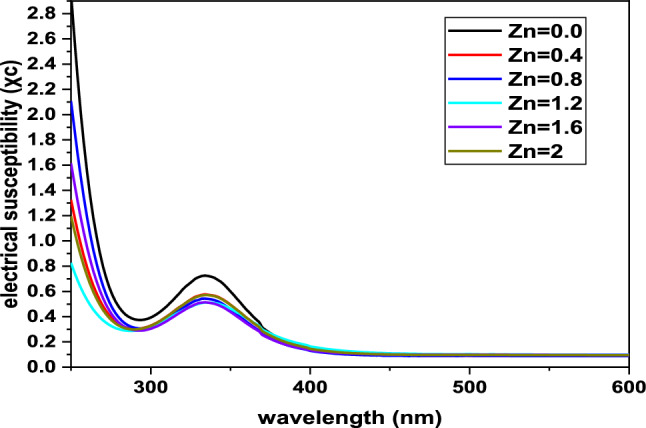
Electrical susceptibility of BaNi_2-x_Zn_x_Fe_16_O_27_ ferrites evaluated via wavelength

At 800 nm, the average electrical susceptibility is approximately 0.0961. Figure 8 presents the relationship between electrical susceptibility (${\chi }_{c}$) and wavelength. As the concentration of Zn increases, the electrical susceptibility decreases, ranging from 0.7 to 0.4. This reduction indicates that higher Zn content in the material reduces its ability to polarize in response to an electric field.

## Conclusion

The Zn concentration in the BaNi_2-x_Zn_x_Fe_16_O_27_ ferrite significantly impacts the optical properties of the material. This impact allows band gap tunability through Zn doping, which is a vital factor in the optical design of ferrite-based electroluminescent devices and other optoelectronic applications. Future work might consider the use of other metal cations in place of Zn to determine their useful band gap tunabilities, resolve the size variance between crystallites and nanorods, and optimize the ferrite for a greater range of technologies.

The important impact of Zn concentration on the optical properties of BaNi_2-x_Zn_x_Fe_16_O_27_ is underscored in this research. Contrasting with what has been frequently reported in the scientific literature, this work shows that incorporating Zn into BaNi_2-x_Zn_x_Fe_16_O_27_ does not blue shift the absorption edge but can instead increase the bandgap energy directly, with the energy difference serving as a useful marker. The band gap tunability is a significant result that makes the BaNi_2-x_Zn_x_Fe_16_O_27_ system potentially useful for a range of optoelectronic applications. Optoelectronic devices work fundamentally by manipulating electron and hole pairs, or excitons, across a bandgap.

Ferrites of the composition BaNi_2-x_Zn_x_Fe_16_O_27_ with x = 0.0, 0.4, 0.8, 1.2, 1.6, and 2.0 were synthesized by the ceramic method. They displayed a single-phase hexagonal structure and, with increasing x, exhibited expansion of the lattice parameters. This expansion results from the larger ionic radius of zinc as compared to that of nickel. Although the synthesis temperatures were kept at the same level for all samples, there were fluctuations in the average crystallite sizes. The smallest size was found for the sample with x = 0.4; the largest, for x = 2.0. These size fluctuations suggest that nickel inhibits while zinc promotes crystallite growth during synthesis.

The band gap, determined through Tauc plots, increased from 2.78 eV to 2.97 eV as Zn concentration increased, indicating potential for band gap engineering in optoelectronic applications. Additionally, the transmittance of each sample increased with wavelength (300–400 nm), reaching a saturation point at longer wavelengths, with strong absorption observed around 334 nm. The absorption edge shifted toward shorter wavelengths with higher Zn content, and the absorption coefficient decreased with increasing wavelength up to around 320–300 nm in the visible spectrum. In the ultraviolet region (λ < 220 nm), the absorption coefficient decreased, which can be attributed to Zn’s absorbing properties.

Furthermore, both the refractive index and extinction coefficient generally increased with higher photon energy and Zn content, suggesting that Zn doping influences the material’s optical response. Determining the relationship between the optical parameters (n, k) and Zn doping is vitally important for assessing the material’s fitness for key roles in application areas, such as optical data-storage devices. Conductivity was one of the areas we examined closely. Peak conductivities, σ, were obtained with no Zn at a reasonable level for usable material. However, as we introduced more Zn into the samples, σ decreased and became less favorable for application purposes. Nonetheless, all the samples demonstrated good photosensitivity across the UV–visible spectrum. We used UV–visible spectroscopy to examine the optical properties of the synthesized nanoparticles and paid special attention to the absorption spectra and the pivotal band gaps.

These data clearly show that Zn doping affects the optical characteristics of BNF nanoparticles. Their absorption spectra were markedly shifted toward higher energies. For the lowest band gap (2.78 eV) calculated for the undoped BNF, the absorption edge was over 334 nm. Such absorption at these energies (even without accounting for the increase in intensity with Zn doping) suggests that as the Zn concentration increased, the electronic structure of the BNF was altered in a way that significantly increased the band gap.

The observed change indicates that Barium-Nickel Ferrite nanoparticles doped with zinc might serve more effectively in applications needing to harness energy, such as devices that convert light into electric power. Zinc doping seems to push the band gap up and, therefore, might help make zinc-doped BNF nanoparticles serve in a greater range than undoped particles in certain electronic applications. The study helps clarify the effect doping has on changing the electronic properties of this type of compound.

## Data Availability

All necessary data generated or analyzed during this study are included in this published article as figures, and other auxiliary data are available from the corresponding author upon reasonable request. Sadiq H. Khoreem.khoreems@yahoo.com.
